# Case Report: Unexpected LAMS obstruction following successful EUS-BD in a patient with pancreatic carcinoma

**DOI:** 10.3389/fsurg.2026.1842089

**Published:** 2026-05-29

**Authors:** Xuxia He, Kun He, Qiang Wang

**Affiliations:** Department of Gastroenterology, State Key Laboratory of Complex Severe and Rare Diseases, Peking Union Medical College Hospital, Chinese Academy of Medical Sciences and Peking Union Medical College, Beijing, China

**Keywords:** case report, endoscopic ultrasound-guided biliary drainage, food impaction, lumen-apposing metal stent obstruction, pancreatic carcinoma

## Abstract

**Background:**

Endoscopic ultrasound-guided biliary drainage (EUS-BD) has become an established alternative for managing malignant biliary obstruction. While recurrent biliary obstruction—commonly due to tumor ingrowth or stent migration following EUS-BD—remains a major clinical challenge, food impaction within lumen-apposing metal stents (LAMSs) has been rarely reported.

**Case presentation:**

A 75-year-old man with unresectable pancreatic carcinoma and duodenal invasion, who had undergone successful EUS-BD with LAMS placement 8 months previously, presented with recurrent obstructive jaundice. Initial imaging indicated no significant tumor progression compared with a follow-up CT performed three months earlier. No tumor ingrowth, overgrowth, or sludge accumulation was observed. Subsequent endoscopy revealed that the LAMS was occluded by food debris. Following endoscopic removal of the impacted food, purulent bile overflowed and a coaxial double-pigtail plastic stent was placed through the LAMS to maintain luminal patency. The patient recovered promptly. In retrospect, the patient reported no dietary restrictions since the previous EUS-BD. At 1-month follow-up, the patient remained asymptomatic, without recurrent jaundice or need for reintervention.

**Conclusion:**

This case highlights food impaction as an unusual but clinically significant cause of recurrent obstructive jaundice following EUS-BD with LAMS. Successful management with endoscopic debridement and coaxial plastic stent placement offers a potential endoscopic strategy for this subgroup of patients. Moreover, this case emphasizes the importance of long-term dietary counseling in patients with indwelling LAMS to prevent this rare but potentially severe complication.

## Introduction

Endoscopic ultrasound-guided biliary drainage (EUS-BD) has increasingly emerged as an effective alternative to endoscopic retrograde cholangiopancreatography (ERCP) for the management of malignant biliary obstruction, particularly when ERCP is not feasible due to anatomical constraints such as duodenal invasion or surgically altered anatomy (1). Lumen-apposing metal stents (LAMSs) are increasingly utilized in EUS-BD, especially in EUS-guided choledochoduodenostomy (EUS-CDS) type, because their design facilitates transmural drainage and minimizes migration risk. Recent meta-analyses have demonstrated that EUS-BD provides comparable efficacy and safety to ERCP for malignant biliary obstruction, but significantly reduces the risk of reintervention, tumor ingrowth or overgrowth, and postprocedure pancreatitis (1, 2). Despite its advantages, recurrent biliary obstruction following EUS-BD remains a major clinical challenge. Previous studies have reported that the incidence of recurrent biliary obstruction after EUS-BD ranges from 10% to 25% (3, 4). The common etiologies of stent dysfunction in this setting include tumor ingrowth or overgrowth, stent migration, and sludge accumulation (1, 3). Among those, LAMS dysfunction secondary to food debris occlusion has rarely been documented in EUS-BD cases. Based on insights from EUS-guided gallbladder drainage cases, potential management of LAMS occlusion includes endoscopic debridement and placement of coaxial double-pigtail plastic stents through the LAMS to maintain luminal patency and prevent recurrent biliary obstruction (5–7).

Herein, we report a case of recurrent biliary obstruction secondary to food impaction within a LAMS after EUS-BD in a patient with unresectable pancreatic cancer. This case highlights an uncommon etiology of stent dysfunction, demonstrates the efficacy of endoscopic reintervention with coaxial plastic stent placement, and underscores the importance of dietary counseling in this patient population.

## Case presentation

The timeline of the clinical course is summarized in Supplementary Table S1. A 75-year-old man was admitted to the emergency department due to abdominal pain persisting for 3 weeks and jaundice for 1 day.

**Figure 1:**
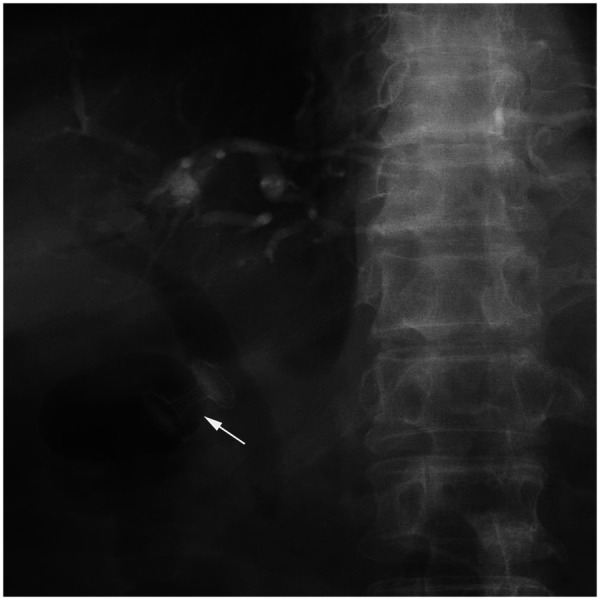
Abdominal X-ray showing a successfully implanted lumen-apposing metal stent (LAMS) (white arrow) between the duodenal bulb and common bile duct. The LAMS has a typical “hourglass” shape with bilateral flanges.

For past medical history, the patient had successfully undergone EUS-BD with LAMS implantation 8 months previously for obstructive jaundice secondary to newly diagnosed unresectable pancreatic carcinoma with duodenal invasion (Figure 1). The LAMS used was an 8 mm × 8 mm electrocautery-enhanced lumen-apposing metal stent (Hot AXIOS, Boston Scientific). The procedure was performed under endoscopic guidance, with direct puncture of the common bile duct from the duodenal bulb, followed by guidewire placement, tract dilation, and LAMS deployment. After EUS-BD, jaundice resolved rapidly. Considering the unresectability of the tumor, the patient underwent chemotherapy and remained stable until the current presentation. Other past medical history and family history were unremarkable.

At the present admission, physical examination revealed severe jaundice and abdominal tenderness, without hepatomegaly or Murphy's sign. Initial vital signs were relatively unstable: temperature 36.2°C; respiratory rate 26 breaths/min; heart rate 129 beats/min; blood pressure 86/56 mmHg; and oxygen saturation 99% on room air. Laboratory tests supported recurrent obstructive jaundice: total bilirubin 109 μmol/L, direct bilirubin 93 μmol/L, and alanine aminotransferase 120 U/L. High blood lactic acid of 7.9 mmol/L was noted, with elevated inflammatory indicators: white blood cells 10.65 × 10⁹/L, procalcitonin 24.89 ng/mL, and hypersensitive C-reactive protein 176.77 mg/L. Based on the presence of fever (although initial temperature was 36.2°C, the patient had documented fever of 38.5°C 2 h before admission), jaundice, abdominal pain, hypotension, and laboratory evidence of systemic inflammation, a diagnosis of acute obstructive suppurative cholangitis (AOSC) was confirmed. Abdomen CT revealed a mass in the pancreatic head invading the descending duodenum, similar to findings on the previous follow-up CT performed 3 months earlier. The implanted LAMS remained *in situ* between the duodenal bulb and common bile duct, with marked dilation of upstream bile ducts. No clear tumor ingrowth/overgrowth or stent migration was seen on CT.

**Figure 2:**
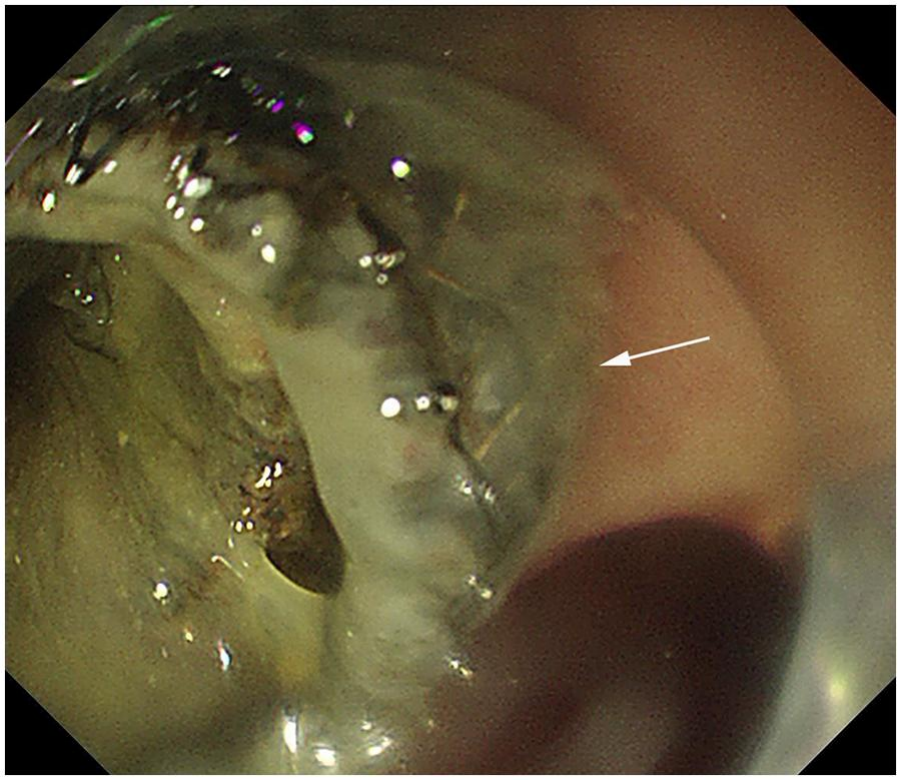
Endoscopic view of the LAMS lumen occluded by impacted food debris (white arrow). No tumor ingrowth or overgrowth was visible at the stent margins.

**Figure 3:**
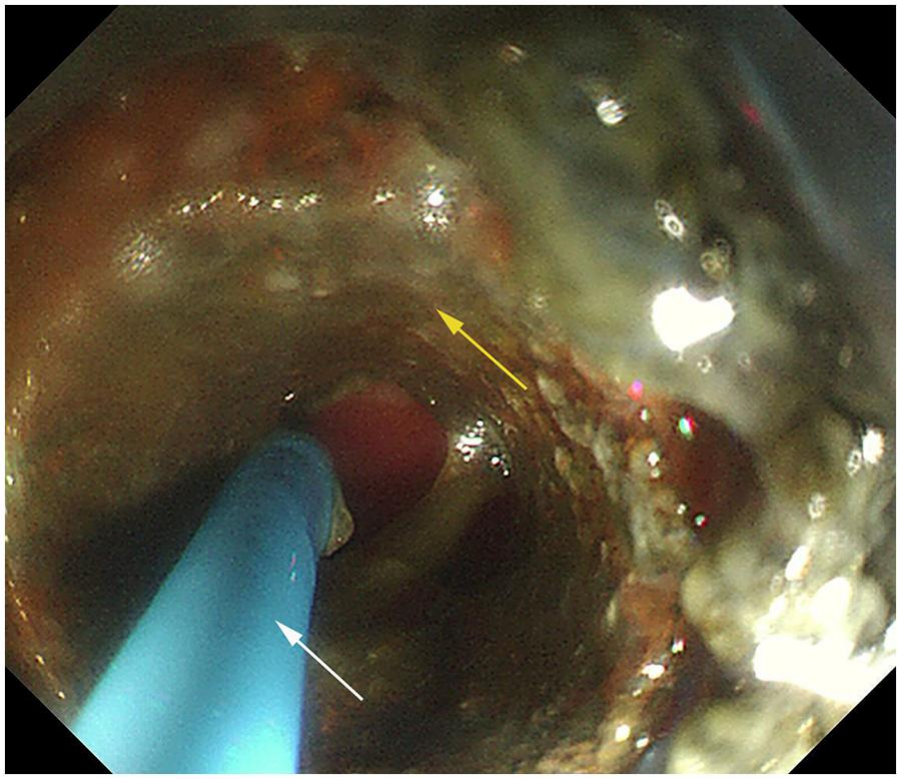
Endoscopic image after removal of food debris: Purulent bile is seen flowing through the LAMS lumen. A double-pigtail plastic stent (white arrow) has been placed coaxially through the LAMS (yellow arrow) to maintain luminal patency.

**Figure 4:**
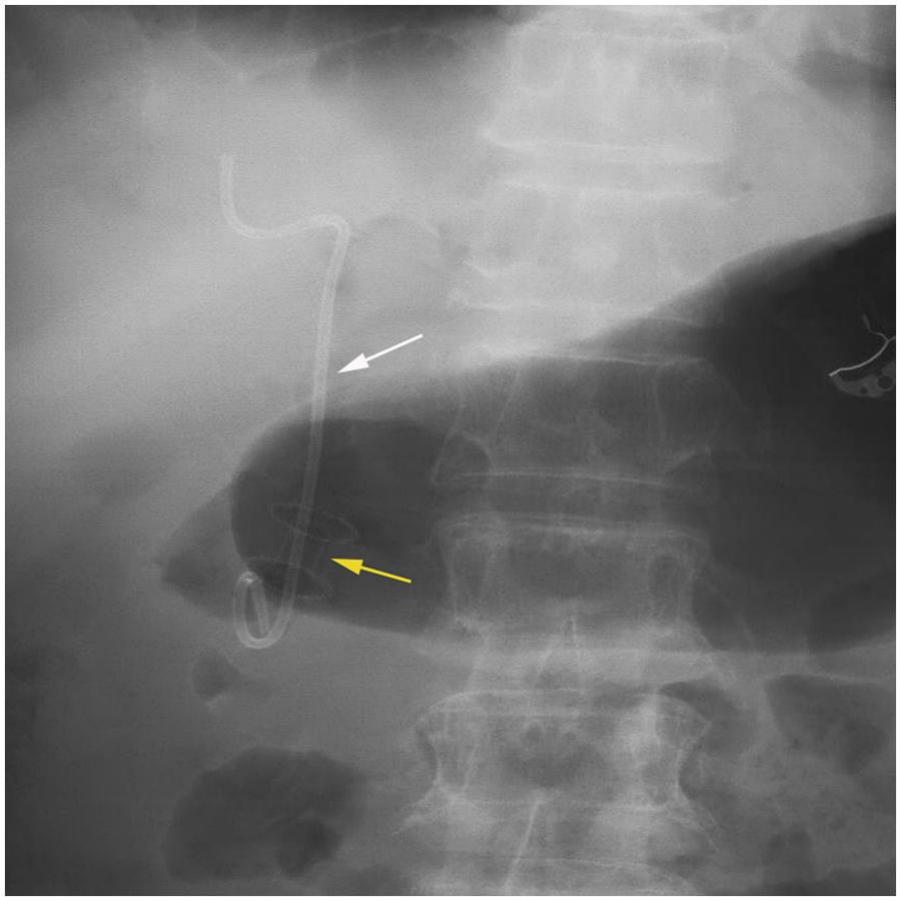
Abdominal X-ray confirming successful placement of a coaxial double-pigtail plastic stent (white arrow) within the LAMS (yellow arrow). The pigtail curls are visible at both ends of the plastic stent.

After initial anti-infective and anti-septic treatment, the patient's vital signs temporarily stabilized. Considering the cause of AOSC, obstruction of LAMS secondary to tumor progression was suspected, and emergency endoscopy was performed. However, endoscopy unexpectedly revealed that the LAMS was occluded by food debris (Figure 2). No tumor ingrowth, overgrowth, or sludge was observed. The LAMS lumen was filled with food debris. Following endoscopic removal of the food using rat-tooth forceps and a retrieval balloon, purulent bile overflowed. Cholangiography was then performed, demonstrating the biliary tract with smooth contrast medium outflow. A coaxial double-pigtail plastic stent (7Fr, 7 cm; Cook Medical) was placed through the LAMS to maintain luminal patency and reduce the risk of recurrent occlusion (Figures 3, 4). No tumor progression was found on subsequent evaluation. On detailed questioning, the patient admitted to having no dietary restrictions since the previous EUS-BD, often eating nuts, raw vegetables, and foods requiring thorough mastication.

After treatment, the patient recovered rapidly, and jaundice resolved. He was discharged on day 7 post-reintervention. At 1-month follow-up, the patient remained asymptomatic, with normal bilirubin levels and no evidence of recurrent obstruction on abdominal ultrasound. No further endoscopic reintervention was required. The patient was referred to oncologists for continued chemotherapy.

## Discussion

Although EUS-BD has shown favorable stent patency compared with alternative approaches such as ERCP (1, 2), recurrent biliary obstruction after EUS-BD has become an increasingly relevant clinical issue as its use expands and patients with the underlying disease survive longer (8). However, literature on reintervention for recurrent biliary obstruction after EUS-BD remains relatively lacking. Here, we report a case of recurrent biliary obstruction due to food impaction within a LAMS placed via EUS-BD 8 months previously in a patient with unresectable pancreatic carcinoma. This case illustrates an unusual mechanism of late stent dysfunction and highlights the importance of dietary counseling for postprocedural management.

Although EUS-BD has been shown to outperform ERCP and percutaneous transhepatic biliary drainage in reducing stent dysfunction, postprocedural pancreatitis, and tumor ingrowth or overgrowth (9), late adverse events remain clinically significant. A large multicenter study of 123 patients undergoing EUS-CDS with LAMS reported a biliary obstruction rate of 16.3% during a mean follow-up of 242 days (10). Notably, the presence of a duodenal stent and a bile duct diameter less than 15 mm were identified as independent risk factors for subsequent obstruction (10). Documented etiologies included tumor ingrowth or overgrowth, stent migration, and sludge accumulation (1, 3). These findings underscore the multifactorial nature of stent dysfunction and the importance of individualized risk assessment.

In our case, these common causes were excluded by CT and endoscopy, while food impaction was identified as the cause. Several factors may have contributed to food impaction. First, the patient had duodenal invasion by the pancreatic tumor, which can impair gastric emptying and duodenal motility, potentially leading to stasis of food particles in the duodenal bulb. Second, the LAMS created a wide-bore direct communication between the duodenal lumen and the biliary tree, facilitating enterobiliary reflux. Third, the patient's poor dietary habits (consuming nuts, raw vegetables, and other high-particulate foods) likely increased the burden of solid debris entering the stent. Fourth, the absence of a coaxial plastic stent at the initial procedure may have allowed food debris to directly occlude the wide LAMS lumen. These factors collectively predisposed to food impaction in this patient. Food impaction as a cause of LAMS occlusion appears exceptionally rare in the biliary tree. While a case of food impaction of LAMS in endoscopic ultrasound-guided gallbladder drainage for acute cholecystitis has been reported (5), similar occurrences following EUS-CDS have, to our knowledge, been scarcely reported. This case therefore represents an unusual cause of stent dysfunction, where endoscopy unexpectedly revealed food debris as the culprit. This highlights the importance of maintaining a broad differential diagnosis when evaluating recurrent jaundice in patients with indwelling LAMS, particularly when imaging does not clearly demonstrate tumor progression.

For management strategies of LAMS dysfunction, endoscopic reintervention via the existing transmural route is technically feasible in most cases, with reported clinical success rates of approximately 80% for desobstruction procedures (10). The therapeutic and prophylactic value of coaxial plastic stents within LAMS has recently been clarified by high-quality evidence. A multicenter randomized controlled trial specifically compared EUS-BD using LAMS alone versus LAMS with coaxial double-pigtail plastic stents in patients with malignant distal biliary obstruction. The coaxial stent group demonstrated a significantly lower rate of recurrent biliary obstruction and shorter hospitalization (7). These findings provide robust evidence supporting the prophylactic value of coaxial plastic stent placement at the time of initial EUS-BD, as well as its therapeutic utility in managing subsequent established occlusion. However, it should be noted that evidence from other LAMS applications, such as drainage of pancreatic fluid collections, has yielded conflicting results regarding the benefit of coaxial stents, suggesting that indication-specific considerations are important (11). In our case, endoscopic removal of impacted food debris resulted in immediate drainage of purulent bile, followed by placement of a coaxial double-pigtail plastic stent through the LAMS to maintain luminal patency. The rapid resolution of jaundice and recovery of the patient indicate the efficacy of this strategy in food impaction of LAMS after EUS-BD.

Moreover, an important and underappreciated aspect of post-EUS-BD care is dietary counseling. The present case revealed that the patient had no dietary restrictions since the previous EUS-BD, implicating poor dietary habits as a contributing factor to stent occlusion. Currently, no established guidelines exist regarding dietary recommendations for patients who have undergone EUS-BD with LAMS placement. However, analogous experiences from EUS-guided gallbladder drainage have prompted calls for dietary counseling (5). Given that LAMS create a direct communication between the gastrointestinal lumen and the biliary tree, the theoretical risk of food debris entering and occluding the stent is biologically plausible. Based on this case, we suggest that clinicians counsel patients on the following measures: thorough mastication of food, avoidance of foods with high particulate content (e.g., nuts, raw vegetables with fibrous components, seeds), and consideration of a low-residue diet. These recommendations should be balanced against nutritional needs, particularly in cancer patients. The present case reinforces the need for such counseling, although evidence supporting these dietary modifications remains scarce, and further studies are required to establish definitive guidelines.

## Conclusions

This case highlights food impaction as an unusual but clinically significant cause of recurrent biliary obstruction following EUS-BD. It underscores the importance of including this etiology in the differential diagnosis of stent dysfunction, particularly when tumor progression is not evident. Successful management with endoscopic debridement and coaxial plastic stent placement supports the efficacy of this approach in EUS-BD patients. Furthermore, this case draws attention to long-term dietary management in patients with indwelling LAMS—an aspect of postprocedural care that needs greater emphasis and further study. As EUS-BD continues to evolve as a primary drainage modality for malignant biliary obstruction, awareness of this manageable complication and appropriate dietary counseling may help reduce the risk of stent dysfunction and improve clinical outcomes.

## Data Availability

The original contributions presented in the study are included in the article/Supplementary Material; further inquiries can be directed to the corresponding author.
