# Supplementary material for: Case Report: Unexpected LAMS obstruction following successful EUS-BD in a patient with pancreatic carcinoma
Source: Front Surg. 2026 May 29;13:1842089. doi: 10.3389/fsurg.2026.1842089 (PMC13262194; doi:10.3389/fsurg.2026.1842089)
Supplement: Supplementary file 1 [file Table1.docx]

**Supplemental Table 1. Timeline of clinical course**

| **Time point** | **Event** |
| --- | --- |
| 2025-03-24 | EUS-BD with LAMS for obstructive jaundice due to unresectable pancreatic cancer with duodenal invasion; jaundice resolved; chemotherapy initiated |
| 2025-07-09 | Follow-up CT: stable disease, no tumor progression |
| 2025-11-13 | Presented with abdominal pain (3 weeks) and jaundice (1 day); unstable vitals; labs: TB 109 μmol/L, DB 93 μmol/L, WBC 10.65×10⁹/L, PCT 24.89 ng/mL; CT: stable tumor, LAMS in situ with dilated bile ducts; diagnosis: AOSC |
| 2025-11-13 | Initial antibiotics and fluids; emergency endoscopy: LAMS occluded by food debris (no tumor ingrowth/overgrowth, no sludge); endoscopic debridement; coaxial double-pigtail plastic stent placed |
| 2025-11-14 | Jaundice resolving; bilirubin decreasing; clinically stable |
| 2025-11-18 | Discharged; jaundice resolved |
| 2025-12-29 | Follow-up: asymptomatic; normal bilirubin; ultrasound: no recurrent obstruction |

EUS-BD, endoscopic ultrasound-guided biliary drainage; LAMS, lumen-apposing metal stent; CT, computed tomography; TB, total bilirubin; DB, direct bilirubin; WBC, white blood cell count; PCT, procalcitonin; AOSC, acute obstructive suppurative cholangitis.
